# Structural Modeling and Analysis of Pregnancy-Associated Glycoprotein-1 of Buffalo (*Bubalus bubalis*)

**DOI:** 10.5402/2012/481539

**Published:** 2012-03-12

**Authors:** Jerome Andonissamy, S. K. Singh, S. K. Agarwal

**Affiliations:** Division of Animal Reproduction, Indian Veterinary Research Institute, Uttar Pradesh, Bareilly 243122, India

## Abstract

The present study was conducted to design and analyze the structural model of buffalo pregnancy-associated glycoprotein-1 (PAG-1) using bioinformatics. Structural modeling of the deduced buffalo PAG-1 protein was done using PHYRE, CONSURF servers and its structure was subsequently constructed using MODELLER 9.9 and PyMOL softwares Buffalo PAG-1 structural conformity was analyzed using PROSA, WHATIF, and 3D-PSSM servers. Designed buffalo PAG-1 protein structure on BLAST analysis retrieved protein structures belonging to aspartic proteinase family. Moreover *in silico* analysis revealed buffalo PAG-1 protein retained bilobed structure with pepstatin-binding clefts near the active sites by docking studies with pepstatin A using PatchDock server. Structural studies revealed that the amino and carboxy terminal containing aspartic residues are highly conserved and buried within the protein structure. Structural conformity studies showed that more than 90% of the residues lie inside favored and allowed regions. It was also deduced that buffalo PAG-1 possesses low and high energy zones with a very low threshold for proteolysis ascertaining the stableness of the buffalo PAG-1 protein structure. This study depicts the structural conformity and stability of buffalo PAG-1 protein.

## 1. Introduction

The pregnancy-associated glycoproteins (PAGs) constitute a large group of proteins belonging to the aspartic proteinase superfamily expressing in the placenta of eutherian mammals. PAGs are acidic glycoprotein sharing more than 50% amino acid sequence identities with Pepsin, Cathepsin D and E [1]. They are secreted from the outer epithelial cell layer of placenti of various ungulate species possessing a predominant role in the placentogenesis, placental modeling, and embryogenesis during pregnancy in domestic species. Many PAG genes have been cloned and identified in many domestic animals such as cattle, sheep, goat, pig, and wild ruminants' species [2–4].

Structural modeling studies in bovine and porcine reveal their bilobed structure and proteolytically inactiveness due to key mutation at the active sites (alanine replaced by glycine: Gly34) residue whose presence would cause displacement of the catalytic water molecule from its normal position between the two catalytic aspartic residues (Asp32 and Asp215). Studies on bovine PAGs show that they have retained the peptide-binding cleft of aspartic proteinases to bind pepstatin and this property is an important factor to characterize members of aspartic proteinase. Moreover PAGs share their identity with other members of aspartic proteinase family, namely, rennin [5, 6].

Recently buffalo pregnancy-associated glycoprotein-1 (PAG-1) gene sequence was deduced and *in silico* analysis revealed buffalo PAG-1 to share more than 80% homology with other members of aspartic proteinase family. Moreover deduced buffalo PAG-1 amino acid sequence also reveals key mutation at the active site which renders it proteolytically inactive. Predicted structure of buffalo pregnancy-associated glycoprotein-1 revealed it to possesses aspartic acid residues near the active site [7]. But structural modeling and its analysis of buffalo PAG-1 protein is still lacking. Considering the paucity of knowledge, the present work was carried out to design and analyze the structural model of pregnancy-associated glycoprotein-1 buffalo (*Bubalus bubalis*). 

## 2. Material and Methods

### 2.1. Buffalo PAG-1 Protein Modeling and Docking

Secondary protein structure prediction from amino acid sequence was performed with PHRYE and CONSURF server [8, 9] to predict the location of the conserved sites in buffalo PAG-1 amino acid sequence. The comparative modeling program MODELER 9.9 [10] in conjunction with PYMOL [11] was done to construct various models of buffalo PAG-1.

### 2.2. Buffalo PAG-1 Protein Docking

Structural docking studies with aspartic proteinase inhibitor, namely, pepstatin A on buffalo PAG-1 protein was carried out using PatchDock [12] server to confirm the affinity of protease inhibitors on buffalo PAG-1 protein.

### 2.3. Buffalo PAG-1 Structural Conformity Analysis

Buffalo PAG-1 structural analysis was carried out with sequences sharing similarity (>25% with respect to the amino acid residue-long sequence of PAG-1) and having similar secondary structure matching with the predicted secondary structure of the target PAG-1 sequence. They were obtained from threading PAG-1 sequence onto known structure by using the PHYRE server. Energy nature, structural conformity, chemical nature, Ramachandran plots, and Z scores of the PAG-1 of the deduced model of buffalo PAG-1 were analyzed using software packages such as PROSA [13], PROCHECK [14], and WHATIF [15]. Multiple alignments of similar structurally related proteins were aligned with their PDB identity using PHYRE software to confirm the chemical nature of buffalo PAG-1. SAPS software was used to predict the potential sites for proteolysis of the PAG-1 protein indirectly measuring the stability of the protein [16–18].

## 3. Result and Discussion

In the present study buffalo PAG-1 was subjected to structural analysis to study the protein model conformation and stability.

### 3.1. Protein Modeling and Docking

**Figure 1:**
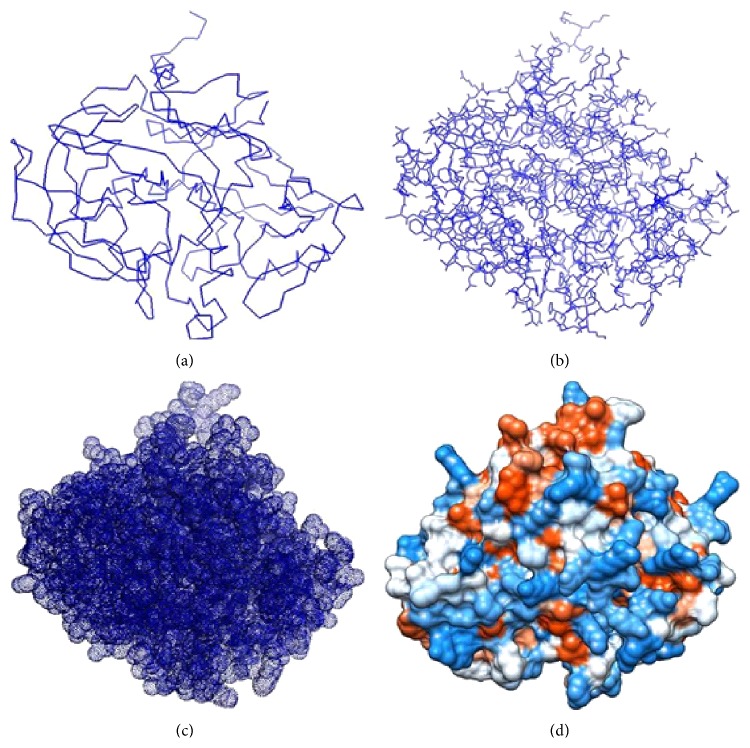
Various models of buffalo PAG-1 protein. (a) Line; (b) ball and stick; (c) mesh; (d) space filling.

Buffalo PAG-1 protein model was designed using software, namely, MODELLER 9.9 and reconstructed using PyMOL. Various models of buffalo PAG-1 were constructed, namely, line, ball and stick, mesh, and space filling model. It is evident from deduced models that buffalo PAG-1 has retained bilobed structure with cleft for pepstatin A binding (Figure 1).

**Figure 2:**
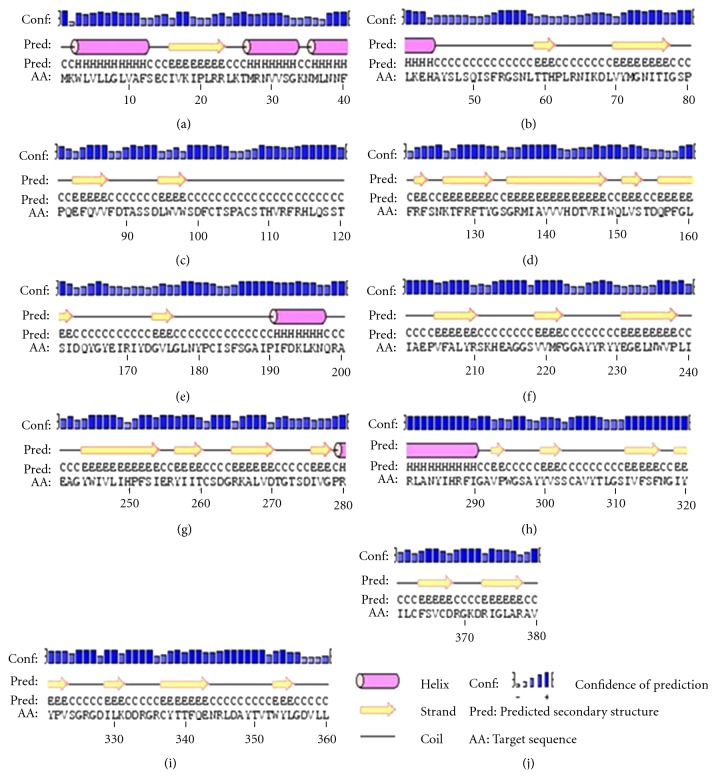
Predicted secondary structure of buffalo PGA-1 protein.

**Figure 3:**
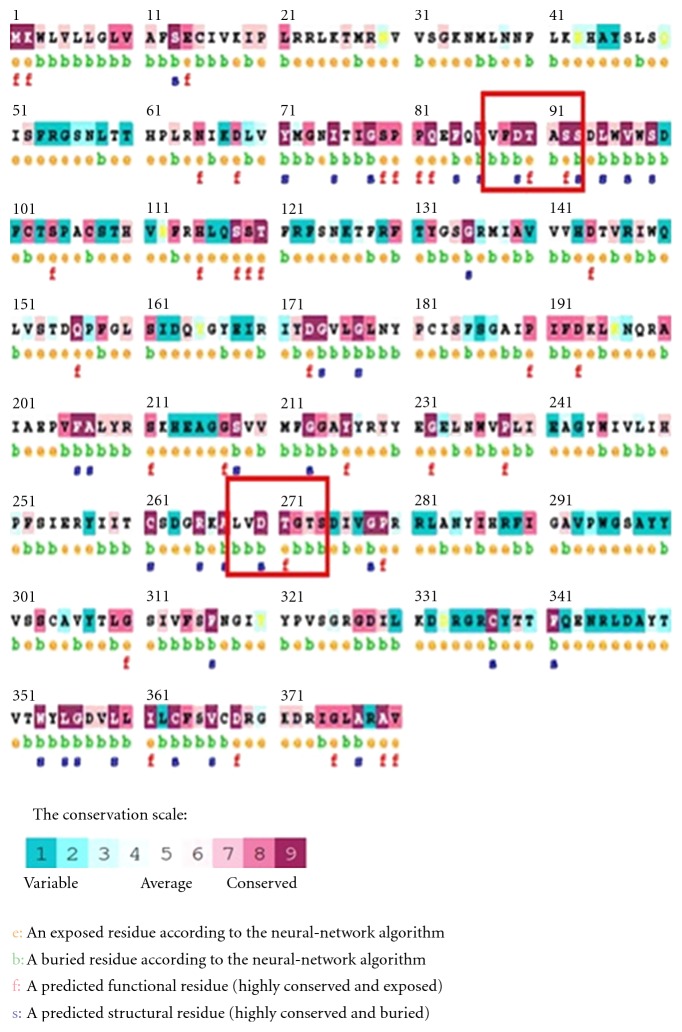
Predicted functional and structural residues in buffalo PAG-1 protein.

Secondary structure prediction of buffalo PAG-1 protein using PHYRE and CONSURF software revealed surface and buried regions of buffalo PAG-1 protein with their respective conformation. The entire buffalo PAG-1 molecule has distinct regions of helices and strands separated with coils. It was deduced that buffalo PAG-1 predicted secondary structure revealed strands and coil in their active sites (85–95 aa) and (270–279 aa) when compared to helices in other regions. The helix conformation was deduced in signal sequence as well as in residues 25–45, 191–199, and 278–290 (Figure 2). Moreover the neural network algorithm in CONSURF server revealed the conserved, buried, surface, and functional residues in buffalo PAG-1 protein. The buffalo PAG-1 protein showed very high degrees of conserved regions in amino and carboxy terminals near the active aspartic regions 85–95 and 268–274 residues confirming its belonging to aspartic proteinase family. Moreover majority of the residues near the active sites were buried inside the protein model and the potential functional residues' locations were predicted in PAG-1 protein molecule (Figure 3).

**Figure 4:**
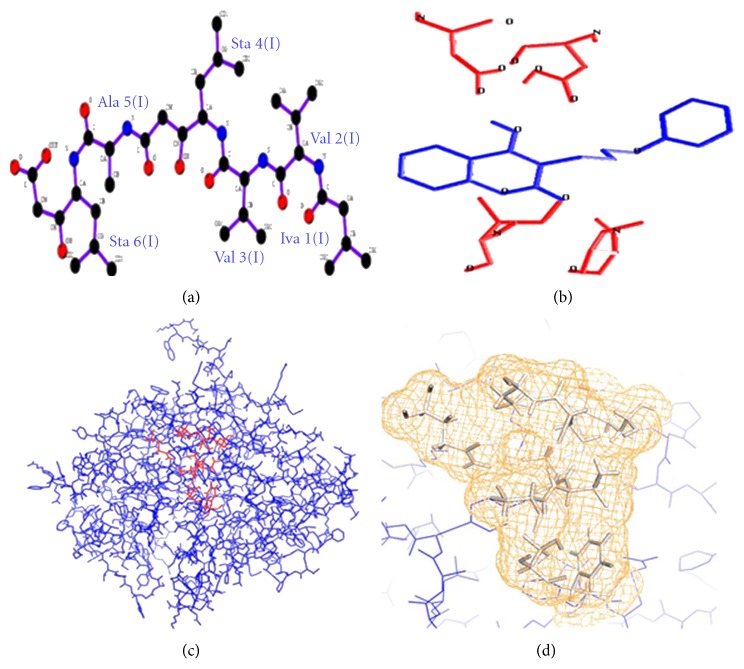
Docking of buffalo PAG-1 with pepstatin A. (a, b) Structure of pepstatin A; (c, d) docking of pepstatin A on buffalo PAG-1 protein.

The predicted protein structure was analyzed with PHYRE server to retrieve similar protein molecules having similar structural conformity. Blast analysis retrieved 50 protein molecules from protein database out of which only 10 molecules which showed close similarity to buffalo PAG-1 were considered for structural analysis. Using PHYRE server many structural similar molecules were retrieved with their unique PDB identity. Molecules having more than 40% identity and showing 100% confidence level were taken into consideration for comparison with buffalo PAG-1 (Table 1). Ten different molecules retrieved from the protein database showed biochemical similarity to members of aspartic proteinase from PHYRE database. It is evident from the structural comparison that all these members of aspartic proteinase family retain the bilobed structure similar to buffalo PAG-1 protein structure. Buffalo PAG-1 protein on docking with specific aspartic proteinase inhibitor, pepstatin A (a hexapeptide consisting of rare amino acids, namely, isovaline and statine: PDB: 1 pso) revealed binding near the active sites in the cleft region between the bilobed structure similar to docking sites reported in bovine and porcine PAG model. These binding studies confirmed the buffalo PAG-1 belonging to aspartic proteinase family. It is also evident that buffalo PAG-1 possesses binding sites for aspartic protease inhibitors (pepstatin A) (Figure 4).

### 3.2. Protein Structure Conformity Analysis

**Figure 5:**
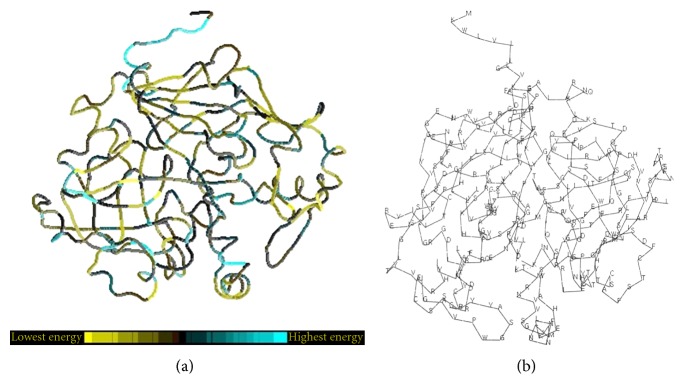
Energy levels corresponding to amino acid residues of buffalo PAG-1 protein.

**Figure 6:**
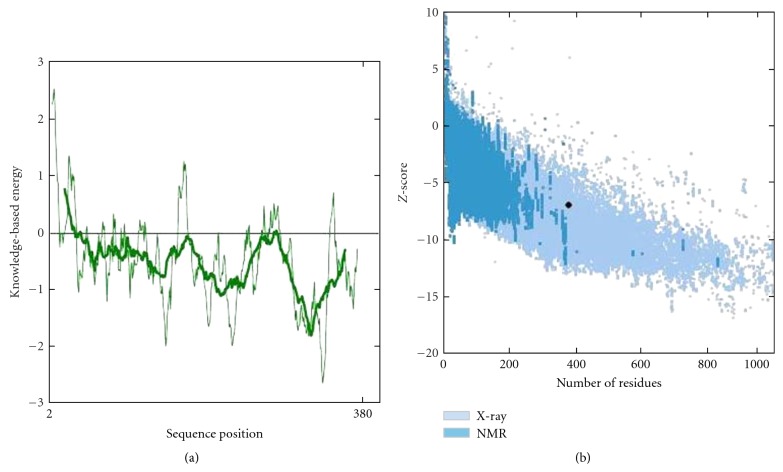
Predicted energy and Z score of buffalo PAG-1 protein.

Analysis with PROSA software revealed the energy status of buffalo PAG-1 in comparison to its various amino acid residues (Figure 5). Energy status of buffalo PAG-1 showed regions of high and low energy. Moreover energy status analysis of the entire molecule revealed the presence of more low energy pockets in comparison to high energy pockets. The active sites in the buffalo PAG-1 protein (85–95) and (275–279) also revealed low energy status revealing the stable structure of the molecule. The Z score of the buffalo PAG-1 protein was −7.01 on analyses in X-ray and NMR methods showing the good quality of deduced buffalo PAG-1 protein model (Figure 6). 

**Figure 7:**
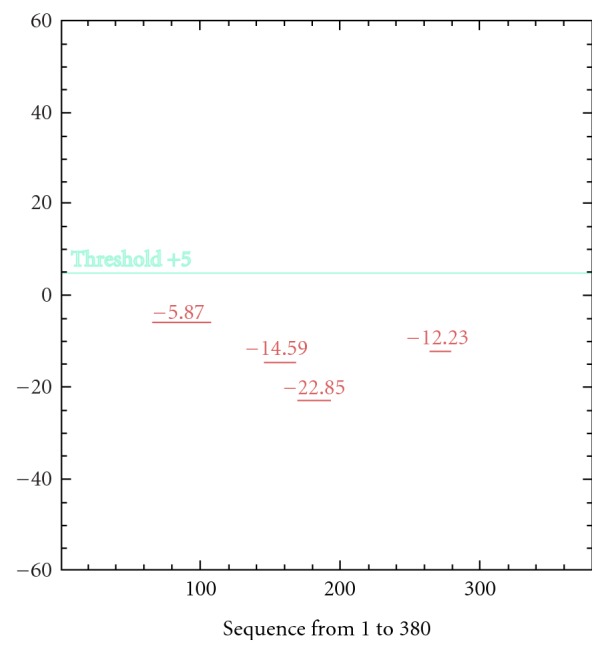
Potential sites and thresholds of proteolysis of buffalo PAG-1 protein.

**Figure 8:**
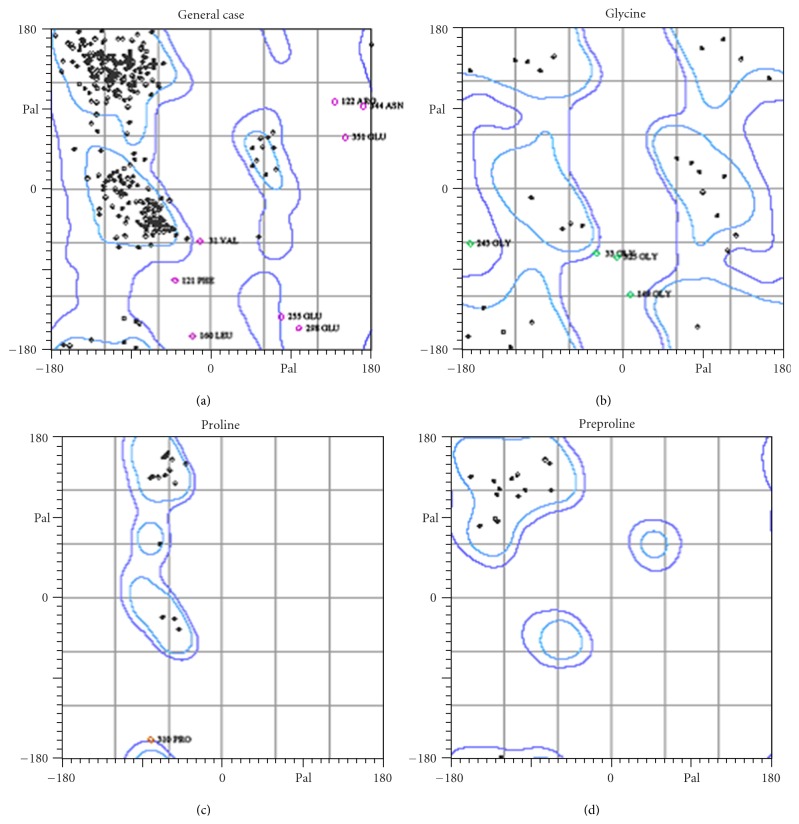
Ramachandran plot of pregnancy-associated glycoprotein-1 of buffalo.

Analysis of buffalo PAG-1 protein using SAPS software revealed potential regions of cleavage by any proteolytic enzyme at residues 8–100, 140–160, 160–180, and 260–280 in the amino acid sequence but it is evident buffalo PAG-1 is stable molecule as the potential sites of proteolysis do not cross the threshold scores (Figure 7). Analysis of Ramachandran plots of buffalo PAG-1 depicted that 89.4% of all residues were located in the favored regions and 96.6% of all residues were located in the allowed regions. There were 13 amino acid residues which were lying outside the favoured or allowed regions (Figure 8). Four glycine residues (33, 149, 243, and 325 aa) and 1 proline residue (310 aa) were outliers in the predicted buffalo PAG-1 protein model. Moreover the predicted centre of mass of buffalo PAG-1 protein is shown in Table 2. Thus structural modeling and analysis of buffalo PAG-1 protein was done and reported for the first time.

## 4. Conclusion

 In conclusion this study reports structural modeling and analysis of buffalo pregnancy-associated glycoprotein-1. Moreover *in silico* analysis of buffalo-associated glycoprotein-1 revealed its bilobed structure with conserved active sites having the property of binding aspartic protease inhibitors thereby confirming its belonging to aspartic proteinase superfamily.

## Figures and Tables

**Table 1:** Structurally related molecules retrieved by PHYRE server.

S.no	PDB identity	Confidence	Identity %	Chemical nature
1	d1psoe	100	42	Pepsin-like
2	c1lywE	99.9	41	Cathepsin D
3	d4pepa	100	40	Pepsin-like
4	d3psga	100	40	Pepsin-like
5	c1tzsA	100	38	Cathepsin E
6	c1avfJ	100	36	Gastricsin
7	c1qdmA	100	34	Prophytepsin
8	c2x0bC	100	28	Renin
9	c2bjuA	100	25	Plasmepsin
10	c3fnuA	100	22	Histo-aspartic protein

**Table 2:** Extremes and approximate center of gravity of buffalo PAG-1 protein model.

S.No	Co-ordinates	Minimum	Maximum	Centre of mass
1	X	−3.206	54.936	26.632
2	Y	−19.376	41.891	11.393
3	Z	−30.298	14.615	−7.661
